# Pulmonary artery hypertension prior to the relapse of adult‐onset Still's disease

**DOI:** 10.1002/rcr2.746

**Published:** 2021-04-28

**Authors:** Yuki Hara, Takayoshi Morita, Katsunao Tanaka, Fusako Sera, Yasushi Sakata, Masayuki Nishide, Yuichi Maeda, Masashi Narazaki, Atsushi Kumanogoh

**Affiliations:** ^1^ Department of Respiratory Medicine and Clinical Immunology, Graduate School of Medicine Osaka University Osaka Japan; ^2^ Department of Cardiovascular Medicine, Graduate School of Medicine Osaka University Osaka Japan; ^3^ Department of Advanced Clinical and Translational Immunology, Graduate School of Medicine Osaka University Osaka Japan

**Keywords:** AOSD, autoimmune disease, PAH

## Abstract

Adult‐onset Still's disease (AOSD) is a rare inflammatory autoimmune disorder characterized by fever, skin rash, and arthralgia. Pulmonary artery hypertension (PAH) rarely occurs with AOSD and has not been reported in the absence of typical symptoms of AOSD. A 33‐year‐old woman was admitted to our hospital with dyspnoea on exertion. Although she had not had symptoms of AOSD for 18 months before her admission, she presented with gradually progressing PAH. Because she had no typical symptoms of AOSD, she was treated with pulmonary vasodilators. However, her PAH did not improve. At one month after vasodilator treatment, she developed a high fever with elevation of ferritin. We determined that her AOSD had relapsed. Immunosuppressants were started and both her AOSD and PAH quickly improved. PAH may develop in the absence of typical symptoms of AOSD and immunosuppressants may be effective in such a case.

## Graphical abstract

We report a patient previously diagnosed with adult‐onset still's disease (AOSD) who presented with pulmonary artery hypertension (PAH) but without typical symptoms of AOSD. Immunosuppressive agents rapidly improved PAH.

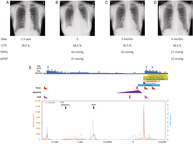

## Introduction

Adult‐onset Still's disease (AOSD) is an autoimmune inflammatory disease [1] and treated with immunosuppressants, such as prednisolone, cyclosporine, and methotrexate. Although some patients may relapse during immunosuppressive treatment or develop serious haemophagocytic syndrome, its prognosis is good. Pulmonary artery hypertension (PAH) is rarely complicated with AOSD. In reported studies, the disease activity of AOSD was high in all patients with PAH complicated with AOSD; however, no studies have shown that PAH developed in the absence of typical symptoms of AOSD. We report the case of a patient with AOSD complicated with PAH initially presenting without the typical symptoms of AOSD.

## Case Report

A 33‐year‐old woman was admitted to our hospital with dyspnoea on exertion. The patient had been diagnosed with AOSD owing to fever, rash, arthralgia, and splenomegaly according to Yamaguchi's criteria at the age of 15 years. Prednisolone was started, and her AOSD had been stable for a long time. Although her AOSD had relapsed two years before admission, the patient had recovered with prednisolone treatment. Eighteen months before admission, she became pregnant and gave birth nine months before admission. During pregnancy, she did not have fever, rash, or dyspnoea. In addition, her C‐reactive protein (CRP) and ferritin levels did not increase. Four months before admission, dyspnoea on exertion gradually appeared without any inflammatory findings. The day before admission, the patient developed fever and dyspnoea.

**Figure 1:**
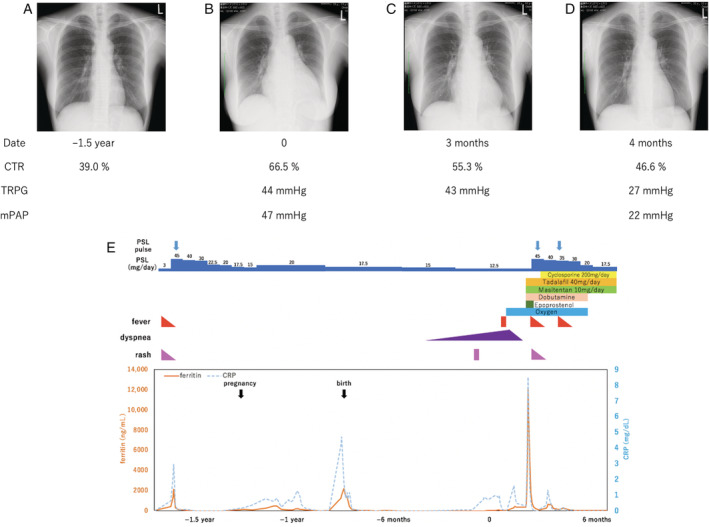
Chest X‐ray of the patient 1.5 years before hospitalization (A), at the time of hospitalization (B), one month after the start of pulmonary vasodilator (C), and one month after enhanced immunosuppressant treatment (D). (E) Clinical course showing the patient's CRP level (blue dashed line) and ferritin level (orange line). Above the graph, symptoms, oxygen dose, pulmonary vasodilators dose, dobutamine, and immunosuppressant dose are described. CRP, C‐reactive protein; CTR, cardiothoracic ratio; mPAP, mean pulmonary arterial pressure; TRPG, tricuspid regurgitation pressure gradient.

Upon admission, the patient's body temperature was 37.8°C. She had no sore throat, swollen lymph nodes, arthralgia, or rash. According to the New York Heart Association classification, the patient was class III. Mild pitting oedema was observed in both lower legs. On blood examination, leucocyte count, CRP, ferritin, N‐terminal pro‐brain natriuretic peptide, and troponin T levels were 6710 cells/μL, 1.01 mg/dL, 32 ng/mL, 4293 pg/mL, and 0.017 ng/mL, respectively. Screening tests for viral infection, such as influenza, hepatitis B virus, hepatitis C virus, human T‐cell leukaemia virus type 1, Epstein–Barr virus, cytomegalovirus, parvovirus B19, and HIV, were negative. Chest X‐ray showed cardiomegaly (Fig. 1A, B). Computed tomography showed no abnormalities other than cardiomegaly and splenomegaly. On electrocardiogram, P waves elevated in II and III, and an abnormal ST change was not detected. On the pulmonary function test, vital capacity and forced vital capacity were normal (86.7% and 91.5%, respectively), but the diffusing capacity for carbon monoxide decreased to 61.5%. On echocardiography, left ventricular ejection fraction was 51% with flattening of the interventricular septum and severe tricuspid regurgitation with a tricuspid regurgitation pressure gradient (TRPG) of 44 mmHg. Right heart catheterization showed that the mean pulmonary artery pressure increased to 47 mmHg with a normal pulmonary arterial wedge pressure (10 mmHg). Cardiac output decreased to 1.98 L/min, resulting in an elevated pulmonary vascular resistance of 18.7 Wood units. No abnormalities were observed on pulmonary perfusion scan. The patient's fever reduced the day after admission without any treatment.

Considering the absence of typical symptoms of AOSD, we could not conclude that the patient's PAH was associated with AOSD. Therefore, oxygen, dobutamine, macitentan, and tadalafil were started without immunosuppressants. One month after these treatments, TRPG remained high at 43 mmHg (Fig. 1C); thus, epoprostenol was started. Two days after the initiation of epoprostenol, a high fever and rash developed. Although epoprostenol was discontinued, her symptoms did not improve. Her ferritin and CRP levels increased to 12,040 ng/mL and 8.53 mg/dL, respectively. We determined that the patient's AOSD had relapsed. Steroid pulse therapy (methylprednisolone (mPSL) 1000 mg/day for three days) with post‐mPSL (36 mg/day) treatment and cyclosporine were started. One month after immunosuppressive treatment with dobutamine, tadalafil, and macitentan, the patient's TRPG decreased to 27 mmHg (Fig. 1D) and a follow‐up right heart catheterization confirmed haemodynamic improvement (Fig. 1E). Finally, the patient was discharged without oxygen and dobutamine. Her PAH and AOSD has remained stable for one year after discharge.

## Discussion

Only 21 case reports have described PAH complicated with AOSD. PAH is a rare complication of AOSD [2, 3]. Connective tissue diseases (CTDs), such as systemic lupus erythematosus (SLE), Sjogren's syndrome (SjS), mixed CTD (MCTD), and systemic sclerosis (SSc), are more likely to complicate PAH. Immunosuppressants are effective on vasculitis that causes PAH complicated with SLE, SjS, or MCTD; however, they are unlikely to be effective on chronic progressive PAH complicated with SSc [2]. Furthermore, the exacerbation of PAH complicated with CTD correlates with the disease activity of CTD. As an important point, PAH rarely develops before the appearance of typical symptoms of CTD [4]. Therefore, deciding whether AOSD without its typical symptoms contributes to the onset of PAH is difficult. However, it should be considered that PAH may precede the appearance of typical symptoms of AOSD, and immunosuppressants may be effective on PAH complicated with AOSD, even in the absence of typical symptoms of AOSD.

AOSD may be triggered by pregnancy. In addition, pregnancy may cause PAH. In a PubMed search, only five cases with postpartum PAH could be detected. Postpartum PAH in two cases was caused by pulmonary artery embolism, and no clear causes for postpartum PAH could be identified in other cases. As a possible mechanism of postpartum PAH, placental hypoxia may induce the production of various vascular mediators in various cells, such as endothelial cells, leading to severe vasoconstriction [5]. In this report, pregnancy may be a possible cause of the onset of PAH complicated with AOSD. Paying attention to the onset of PAH in pregnant patients with AOSD may be necessary.

### Disclosure Statement

Appropriate written informed consent was obtained for publication of this case report and accompanying images.

### Author Contribution Statement

Takayoshi Morita and Yuki Hara designed this report and wrote the manuscript. Katsunao Tanaka and Fusako Sera performed cardiovascular examinations and prescribed vasodilators for PAH. Masayuki Nishide, Yuichi Maeda, Masashi Narazaki, and Atsushi Kumanogoh participated in the discussion. Atsushi Kumanogoh supervised this report. All authors were involved in drafting the manuscript and approved the final version of the manuscript.
